# Employing Nanotechnology to Enhance Ethanol Production in Two *Synechococcus elongatus* Strains

**DOI:** 10.1002/cbic.70480

**Published:** 2026-07-24

**Authors:** Alexandra M. Schirmacher, David A. Russo, Alexandra C. U. Furch, Julie A. Z. Zedler

**Affiliations:** ^1^ Synthetic Biology of Photosynthetic Organisms Matthias Schleiden Institute for Genetics Bioinformatics and Molecular Botany Friedrich Schiller University Jena Jena Germany; ^2^ Bioorganic Analytics Institute for Inorganic and Analytical Chemistry Friedrich Schiller University Jena Jena Germany

**Keywords:** biosynthesis, cyanobacteria, encapsulation peptide, enzymes, ethanol, nanotechnology, synthetic biology

## Abstract

Cyanobacteria have emerged as promising photosynthetic cell factories. However, low product yields remain a bottleneck to industrial exploitation. Here, we use bacterial microcompartment technology to improve product titres using ethanol biosynthesis as the model pathway. Two strategies compared to unmodified enzyme production were tested: first, targeting enzymes to nanofilaments by fusion with encapsulation peptides (EPs) which form non‐covalent interactions with the filaments. Second, the enzyme‐EP fusions were used in the absence of nanofilaments, as EPs tend to form soluble aggregates. Both approaches were tested in *Synechococcus elongatus* PCC 7942 and the fast‐growing *Synechococcus elongatus* UTEX 2973 to validate if fast growth is additionally beneficial for bioproduction. The EPs had the most dramatic effect on enzyme levels and ethanol production leading to an increase in titres of up to 84 times in comparison to the unmodified enzymes. While Se7942 exhibited higher titres per cell, titres were the highest in Se2973 due to a 79% increase in final cell density. Ultimately, the best strategy (enzyme‐EP fusion in the absence of PduA*) led to 1.31 g L^−1^ of ethanol in 6 days. This work demonstrates that nanotechnology‐based strategies can aid cyanobacterial bioproduction and will serve as a blueprint for future engineering efforts.

## Graphical abstract

Here, we report on how bacterial microcompartment technology can be used to improve bioproduction in cyanobacteria. Fusion of encapsulation peptides to the two enzymes of the ethanol biosynthetic pathway increased ethanol titres up to 84 times. 1.31 g L^−1^ of ethanol was produced in 6 days.

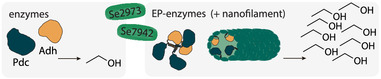

## 1 Introduction

Cyanobacteria are photoautotrophic bacteria that can use carbon dioxide as sole carbon source and sunlight as energy source. They do not require soil for cultivation or refined sugars as substrate and hence do not compete with food production [1, 2]. For these characteristics, they hold great potential as production platforms for a variety of chemicals. Typically, studies aiming to develop cyanobacteria as production platforms have employed classic models such as *Synechocystis* sp. PCC 6803 (hereafter Syn6803) and *Synechococcus elongatus* PCC 7942 (hereafter Se7942). However, these model organisms suffer from relatively slow growth rates and low product titres ranging in mg per L or low g per L scale [2, 3, 4, 5]. More recently, several fast‐growing strains with doubling times of 1.5–2 h have been described including *Synechococcus elongatus* UTEX 2973 (hereafter Se2973) [6] and *Synechococcus* sp. PCC 11901 [7]. Fast growth is considered advantageous for product formation [5, 8]. However, questions have been raised regarding resource allocation between biomass and product formation in fast‐growing strains [5, 9, 10].

One engineering strategy to increase product titres in cyanobacteria is the co‐localisation of the enzymes of a biosynthetic pathway. Co‐localisation has the potential to improve channelling of intermediates and prevent accumulation of toxic intermediates. Traditionally, co‐localisation was often achieved by enzyme fusion [11]. However, recent advances in nanotechnology have shown that biosynthetic pathways can be encapsulated in or attached to DNA‐ and protein‐based structures. Through this approach, significant increases in product titres in heterotrophic production hosts have been achieved [12, 13]. In phototrophic hosts, the use of bacterial microcompartment (BMC)‐based nanotechnology for scaffolding of biosynthetic pathways is still a nascent field [14, 15]. In this context, we have recently established proteinaceous nanofilaments in Syn6803 and Se2973. These nanostructures were composed of the self‐assembling BMC‐protein PduA* and shown to form large, mechanically stable, intracellular filaments to which single proteins can be targeted using short ‘encapsulation peptides' (hereafter EPs) [16].

**FIGURE 1:**
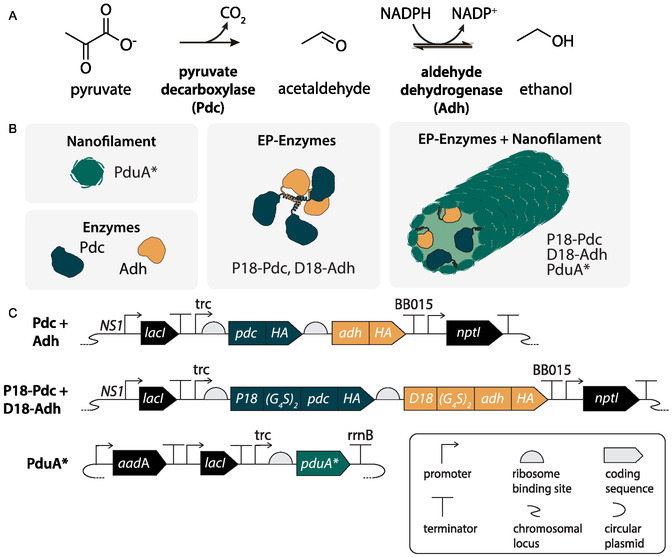
Overview of the ethanol biosynthetic pathway and co‐localisation design. (A) Schematic representation of the chemical reaction catalysed by pyruvate decarboxylase and aldehyde dehydrogenase for ethanol synthesis. (B) Graphical overview of strategies for enzyme‐co‐localisation tested in this study using encapsulation peptides (EPs) and potential arrangement of PduA* nanofilaments. (C) Representation of engineered strain constructs using SBOL visual version 3.0 glyphs [17]. *aadA*: Spectinomycin and Streptomycin resistance cassette, *adh*: aldehyde dehydrogenase, *D18* + *P18*: EPs, (G_4_S)_2_: flexible double 4xglycine‐serine linker, *lacI*: LacI repressor gene, *nptI*: Kanamycin resistance cassette, *HA*: HA‐epitope tag encoding sequence, NS1: neutral site 1, *pdc:* pyruvate decarboxylase, pduA*: PduA derivative with C‐terminal extension [18].

In this study, we aimed to use nanotechnology to co‐localise the two enzymes for ethanol biosynthesis, a pyruvate decarboxylase (Pdc) and an aldehyde dehydrogenase (Adh) (Figure 1A), to enhance ethanol formation. We chose the two closely related cyanobacterial strains, Se7942 and Se2973, as engineering targets. Se7942 is a long‐standing model organism with minimum doubling times of 4.9 h whereas Se2973 is a fast‐growing cyanobacterium with doubling times as short as 1.5 h [6, 19]. Curiously, their genomes are 99.8% identical but a direct comparison of productivity between these strains in the same study is currently lacking.

Two co‐localisation strategies were employed (Figure 1B). First, Pdc and Adh were fused with EPs in the absence of the nanofilaments. EPs have been shown to have self‐aggregating properties; therefore, they possess an inherent potential for co‐localisation [20, 21, 22]. Second, Pdc and Adh were fused with EPs in the presence of the nanofilaments where they are expected to interact with the nanofilament forming protein, PduA*, thus scaffolding the enzymes [23, 24, 25, 26, 27, 28].

Our results ultimately show that use of EPs was sufficient to boost ethanol biosynthesis and increase titres up to 84 times. The work presented here provides new tools for co‐localising biosynthetic pathways in cyanobacteria and demonstrates that nanotechnology can aid the development of cyanobacterial biotechnology.

## 2 Results

### 2.1 EP‐Enzyme Localisation Coupled to Fast‐Growth Dramatically Increased Ethanol Production Titres

To produce ethanol in cyanobacteria, we chose the well characterised biosynthetic pathway of pyruvate decarboxylase (Pdc) from *Zymomonas mobilis* and an aldehyde dehydrogenase (Adh) from Syn6803 (Figure 1A). We then employed elements of BMC technology to modify the pathway with the goal of improving enzyme stability and substrate channelling (Figure 1B). In the EP‐enzyme approach, small EPs were fused to Pdc and Adh. Fusion of EPs to biosynthetic enzymes has been shown to lead to the formation of large active enzyme aggregates [21, 22]. In the EP‐enzyme with nanofilament approach, a nanofilament was added to the strain with the EP‐tagged enzymes to scaffold the biosynthetic pathway. Structural models suggest different PduA filament arrangements [29, 30]. Experimental and modelling evidence suggests that the EP interacts with the hydrophobic groove between PduA hexamers on the convex side [31] which would potentially result in a luminal orientation of P18‐Pdc and D18‐Adh (Figure 1B). Nevertheless, EPs might also influence self‐assembly of PduA* and could lead to formation of other structures beyond nanofilaments [32].

To test the different approaches, *pdc* and *adh* were integrated into the chromosome of Se7942 and Se2973 at neutral site 1 (NS1), either fused only with a C‐terminal HA‐tag (construct Pdc + Adh) or additionally with N‐terminal EPs P18 and D18 (construct P18‐Pdc + D18‐Adh) (Figure 1C). The synthetic operon was placed under the control of an IPTG‐inducible *trc* promoter. Additionally, strains with an antibiotic cassette (*nptI*) integrated into NS1 and a self‐replicative pDF‐trc [33] plasmid without a gene of interest served as a negative control. Successful transformation and segregation were confirmed by colony PCR (Figure S1) as well as sequencing of the PCR‐amplified genomic insertion. To generate strains where Pdc and Adh were targeted to the PduA* nanofilament, pduA* was co‐expressed from a self‐replicating plasmid, as previously reported [16], in a P18‐Pdc + D18‐Adh background strain (Figure 1C).

**FIGURE 2:**
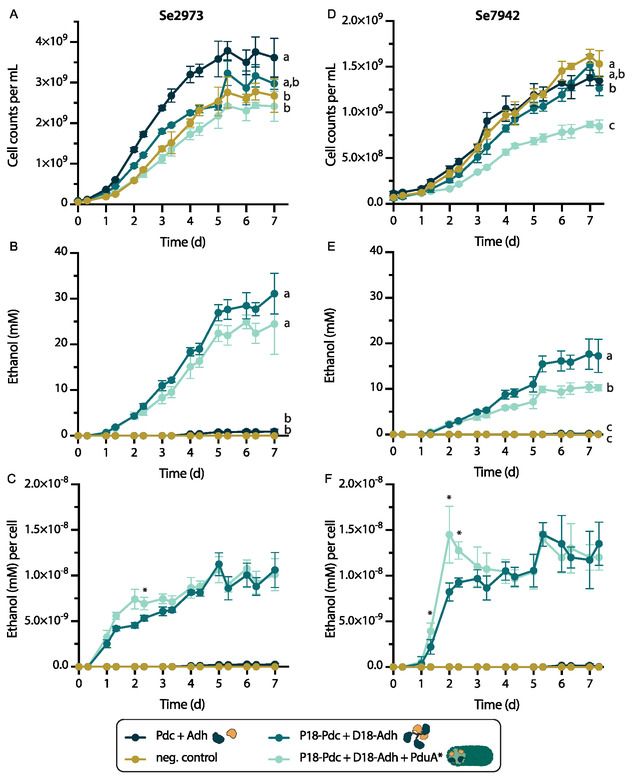
Growth and ethanol production over time of engineered Se2973 and Se7942 strains. Cell counts per mL of Se2973 (A) and Se7942 (D). Total ethanol in the cell‐free supernatant of Se2973 (B) and Se7942 (E) as measured by a colorimetric assay. Ethanol titres normalised to cell counts for Se2973 (C) and Se7942 (F). For all experiments, cultivation was done with a light ramp in the first 48 h from 80 to 300 μmol photons m^−2^ s^−1^ for Se7942 strains, and from 80 to 500 μmol photons m^−2^ s^−1^ for Se2973 strains, followed by constant illumination until the end of the experiment. *n* = 4, error bars ± SD. Significance letters represent statistical comparisons among values on Day 7. These were determined using a one‐way ANOVA followed by a Tukey's multiple comparison test. Significance stars represent statistical comparisons among normalised ethanol titres in early exponential growth phase. These were determined with multiple Student's *t*‐tests and a false discovery rate correction of 0.05.

To determine growth profiles and ethanol formation, a production experiment was setup with the generated Se2973 and Se7942 engineered strains. Se2973 has been shown to achieve maximum growth rates at 38 °C. However, in preliminary tests, ethanol concentrations were found to be higher at 30 °C (Figure S2). Therefore, all experiments were conducted at 30 °C. Based on logistic curves fitted to the growth data of each strain, Se2973 strains exhibited higher growth rates and maximum densities (Figure 2A,D; Table 1). In both Se2973 and Se7942, the strains producing PduA* displayed the lowest growth rates. This is in agreement with our previous observations where expression of PduA* also impacted growth [16]. In addition, interference with cell division was observed resulting in a filamentous phenotype (Figure S3).

**TABLE 1:** Ethanol titres and parameters derived from fitting logistic curves to the Se2973 and Se7942 growth data.

Host	Strain	*r*	*K*	RSDR	EtOH, mM
Se2973	Negative control	1.29	2.66 × 10^9^	8.25 × 10^7^	0 ± 0
	Pdc + Adh	1.41	3.55 × 10^9^	1.40 × 10^8^	0.93 ± 0.37
	P18‐Pdc + D18‐Adh	1.46	2.45 × 10^9^	5.98 × 10^7^	28.49 ± 2.87
	P18‐Pdc + D18‐Adh + PduA*	1.00	2.40 × 10^9^	6.21 × 10^7^	24.95 ± 1.51
Se7942	Negative control	0.82	1.68 × 10^9^	4.89 × 10^7^	0 ± 0
	Pdc + Adh	0.90	1.40 × 10^9^	6.07 × 10^7^	0.21 ± 0.25
	P18‐Pdc + D18‐Adh	0.90	1.37 × 10^9^	5.45 × 10^7^	17.65 ± 3.36
	P18‐Pdc + D18‐Adh + PduA*	0.79	9.35 × 10^8^	3.55 × 10^7^	10.42 ± 1.14

**Note:** *r—*growth rates, *K—*carrying capacity, RSDR—robust standard deviation of the residuals, EtOH (mM)—final concentrations of ethanol measured in the supernatant.

Regarding ethanol production, the strains with the unmodified Pdc + Adh biosynthetic pathway accumulated low amounts of product (<1 mM) despite exhibiting some of the highest growth rates and maximum cell densities (Figure 2B,E; Table 1). Strikingly, when Pdc and Adh were fused to EPs, the ethanol levels increased up to 31 times in Se2973 (up to 28.5 mM) and up to 84 times in Se7942 (up to 17.6 mM) (Table 1). This resulted in maximum daily titres of 4.75 mM d^−1^ for Se2973 and 2.5 mM d^−1^ for Se7942. The addition of PduA* to the P18‐Pdc + D18‐Adh strains led to slightly decreased final ethanol titres, although this difference was only significant for Se7942 (Student's *t*‐test, *p* < 0.001). Nevertheless, when analysing ethanol titres per cell, the presence of PduA* resulted in significantly higher per cell productivity in the early exponential growth phase (Figure 2C,F). This was the case for Day 2.33 (*p* = 0.01) for Se7942 and Days 1.33–3 (*p* = 0.01–0.02) for Se2973. Also notable is the higher productivity per cell of Se7942 (up to 1.5 × 10^−8^ mM ethanol per cell) in comparison to Se2973 (up to 1.1 × 10^−8^ mM ethanol). This suggests that, in some cases, the advantage of using fast‐growing strains for bioproduction may derive from faster biomass accumulation rather than higher per cell productivity. Finally, to assess the health of the strains, chlorophyll *a* and carotenoids were extracted and quantified (Figure S4). In Se2973, despite the modest difference in carrying capacity, chlorophyll *a* values were three times lower in the strain additionally producing PduA* when compared to strain containing only the EP‐fused enzymes. The trend was similar in Se7942. While we observed only a 32% drop in carrying capacity with the addition of PduA*, chlorophyll *a* values were 2.5 times lower. This agrees with our previous study where we observed a significant decrease in chlorophyll *a* and phycocyanin levels in a Se2973 strain producing PduA* [16].

### 2.2 Fusion With EPs Leads to a Dramatic Increase in Pdc Levels

**FIGURE 3:**
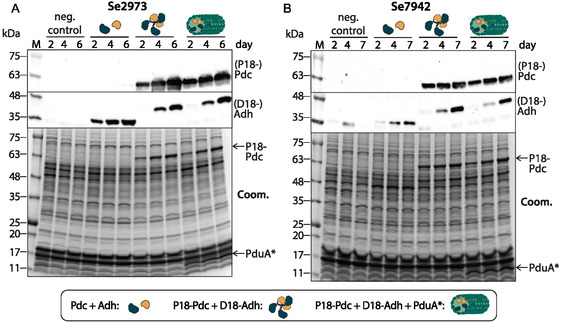
Detection of Pdc and Adh variants at early (Day 2), exponential (Day 4), and stationary (Day 6 or 7) phase in cleared cell lysates of (A) Se 2973 and (B) Se7942. Pdc and Adh variants were detected by immunoblotting using an Anti‐HA antibody. Due to the difference in heterologous protein abundance, different amounts were loaded for immunoblotting. For Pdc detection, 5 µg of protein was loaded for the P18‐Pdc + D18‐Adh and PduA* strains in both Se2973 and Se7942. For the Se2973 negative (neg.) control and Pdc + Adh strain, 30 µg of protein was loaded. For the Se7942 neg. control and Pdc + Adh strain, 25 µg of protein was loaded. For Adh detection, 20 µg of protein was loaded for all samples. For detection of PduA*, and as a loading control, a Coomassie‐stained polyacrylamide gel (Coom.) with 7.5 µg of protein loaded for all samples is shown. Representative western blots are shown. Please note that Pdc is present but only visible upon overexposure of the blot.

We then used immunoblotting to investigate protein levels in all strains at early, exponential, and stationary phase (Days 2, 4, and 6 for Se2973 and Days 2, 4, and 7 for Se7942). In Se2973, levels of unmodified Pdc were below the detection limit in early exponential phase, but a faint band can be seen on Days 4 and 6 (Figure 3A). Unmodified Pdc levels were not visible in Se7942 but a faint band could be detected after overexposure (Figure 3B). Well‐defined bands were visible for unmodified Adh in both strains with a general trend of increasing proteins levels across the three timepoints (Figure 3). Interestingly, when Pdc was fused with EPs a substantial increase in protein levels was observed in Se2973 (Figure 3A) and Se7942 (Figure 3B). Differences in Adh levels with and without EPs were moderate but with a stronger increase of protein in the stationary phase when fused with the D18 EP. The co‐production of PduA* for nanofilament formation led only to minor changes in P18‐Pdc or D18‐Adh levels.

**FIGURE 4:**
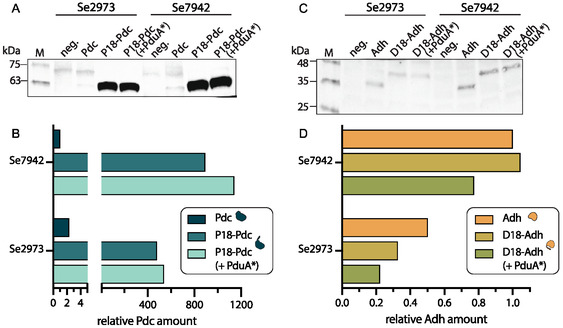
Comparison of Pdc and Adh amounts in ethanol‐producing Se2973 and Se7942 strains. (A) Representative blot of Pdc and P18‐Pdc detected by immunoblotting (anti‐HA). (B) Relative quantitation of Pdc and P18‐Pdc in Se2973 and Se7942 by densitometry. Quantities are normalised to the amount of unmodified Pdc detected in Se7942. (C) Representative blot of Adh and D18‐Adh detected by immunoblotting (anti‐HA). (D) Relative quantitation of Adh and D18‐Adh in Se2973 and Se7942 by densitometry. Quantities are normalised to the amount of unmodified Adh detected in Se7942. Neg.: negative control strain.

To directly compare protein levels in Se2973 and Se7942, cleared lysates from Day 4 were analysed on the same immunoblot. Without EPs, Se2973 produced approximately double the amount of Pdc than Se7942 (Figure 4A,B). Fusion of the EP P18 resulted in a dramatic increase in Pdc levels compared to the unmodified enzyme in both strains (Figure 4A,B). However, the amount of P18‐Pdc observed in Se2973 was only half of that in Se7942 (Figure 4B). When comparing protein levels of unmodified Adh with those fused to EPs, the changes were less dramatic (Figure 4C). But the direct comparison between Se2973 and Se7942 shows that Adh levels in Se7942 were two to three times higher throughout the experiment (Figure 4D).

### 2.3 Substrate Availability Was Not the Limiting Factor During High‐Titre Ethanol Production

Based on the immunoblot analysis, we reasoned that the strong increase in P18‐Pdc compared to Pdc levels relieved a pathway bottleneck posed by this enzyme. Thus, resulting in the strong increase in ethanol titres. To investigate if ethanol production could be further enhanced through increased substrate pools, cultures were supplemented with 2 mM of the reaction substrate pyruvate (Figure 1A) every 24 h. Strikingly, all strains with EP‐tagged enzymes bleached within 2–3 days. Meanwhile, the cultures with unmodified enzymes and all the cultures without supplemented pyruvate retained a healthy green colour (Figure S5A,B). In addition, ethanol titres throughout the experiment were always lower in cultures supplemented with pyruvate (Figure S5C,D). This suggested that the dramatic increase of Pdc may have led to an increased production of the toxic intermediate acetaldehyde.

To verify this hypothesis, we quantified acetaldehyde in the supernatant of the ethanol‐producing strains at 48 h. We observed that under the pyruvate feeding regime acetaldehyde levels were approximately three to seven times higher in all strains (Figure S5E,F). In accordance with the observed toxicity in the growth experiment, the strains with EP‐tagged enzymes produced the highest values of acetaldehyde (Figure S5E,F) which is likely to have become toxic at levels in the range of 150–200 µM. For future experiments, lowering the amount of pyruvate (e.g., 1 mM per day) might help to balance intermediate toxicity with final ethanol output.

Next, to obtain a measure of pathway efficiency, the product‐to‐intermediate (ethanol/acetaldehyde) ratio was calculated. In both Se2973 and Se7942, the ethanol/acetaldehyde ratio was lower during pyruvate feeding which points to an increase in pathway inefficiency (Figure S5G,H). This agrees with the observed decrease in ethanol titres in these conditions. Interestingly, across all experiments the strains with EP‐tagged enzymes consistently showed higher ethanol/acetaldehyde ratios (up to 13 times) when compared to the strains with the unmodified enzymes (Figure S5G,H). While elevated ratios may suggest improved channelling, the increase in Pdc production and stability could be a main reason leading to of this effect. Nevertheless, it demonstrates a clear advantage of using the EP‐tagged enzyme variants over the unmodified pathway.

## 3 Discussion

In this study, we tested BMC technology to improve titres of ethanol biosynthesis in cyanobacteria. We also used this as a platform for a direct comparison of the fast‐growing, biotechnologically relevant Se2973 and the closely related, slower growing, model organism Se7942. The most effective strategy was the fusion of EPs to Pdc and Adh which led to an increase in ethanol titres of up to 84 times in comparison to the unmodified enzymes. Interestingly, Se7942 achieved a 36% higher maximum ethanol titre per cell (1.5 × 10^−8^ mM in comparison to 1.1 × 10^−8^ mM for Se2973). However, by virtue of a 79% increase in final cell density, maximum ethanol titres were higher in Se2973 (28.5 mM, 1.31 g L^−1^) than in Se7942 (17.6 mM, 0.81 g L^−1^).

The higher per cell productivity in Se7942 is likely linked to the observed differences in the levels of Pdc and Adh, which were approximately double of those in Se2973 (Figure 4). These were unexpected observations given that Se2973 has higher amino acid content, CO_2_ fixation rates, and NADP^+^ and ATP production when compared to Se7942 [19, 34, 35]. Previous studies, however, have suggested that the Se2973 circadian clock master regulator *rpaA* is mutated and, consequently, cells are trapped in subjective dawn, programmed to divide continuously [35]. Therefore, this could lead to selective upregulation of gene expression required for fast growth and may ultimately result in limited capacity for heterologous enzyme production.

Mathematical models have hypothesised that a more productive strain would display slower growth rates in comparison to strains that maximise growth rate due to differing proteome allocations. In particular, models predict that strains optimised for growth have higher expression levels of the photosynthetic machinery [9]. Accordingly, in comparison to Se7942, Se2973 exhibits a 1.6‐fold increase in PSI content, a 1.5‐fold increase in cytochrome b_6_f content, and a 2.4‐fold increase in plastocyanin content on a per cell basis [35, 36]. Overall, slower growth seems to be beneficial for heterologous enzyme production and should be coupled with high cell densities for maximal production. Ideally, growth and production phases might need to be separated in a biotechnological production process. This was previously attempted by arresting cell growth through CRISPRi [37, 38]. However, growth arrest also caused a drop of CO_2_ fixation to 25% of the initial rates 2 days after complete growth arrest occurred.

A striking observation was the large increase in ethanol titres when Pdc and Adh were fused with EPs regardless of the presence of nanofilaments. A similar observation was made for the biosynthesis of 1,2‐propanediol where strains producing enzymes tagged with the P18 and D18 EPs exhibited the highest product titres [21]. Here, we observed that only Pdc levels were significantly increased when fused with P18. It is possible that the N‐terminal fusion of P18 changed the folding energy of the mRNA and thereby enhanced the efficiency of the translation initiation [39]. This is a phenomenon that has been observed for multiple peptide tags in *E. coli* [40, 41]. However, this alone does not fully explain the observed changes in protein abundance upon fusion with P18. In our previous work, we only observed higher P18‐mCitrine abundance in the absence of PduA* [16]. In contrast to this, in this work EP fusion to Pdc led to substantially higher protein abundance also in the presence of PduA* (Figures 3 and 4). This suggests additional factors may be contributing to increased protein levels.

In the presence of the nanofilaments, we observed a peak in productivity at 48 h for both Se973 and Se7942. In the early stages of the experiment, when the absolute enzymes levels are not saturating, co‐localisation may have had a positive effect on metabolite channelling, thereby reducing the accumulation of the toxic intermediate acetaldehyde. From there onwards, it is likely that the metabolic burden of maintaining these large intracellular structures had a negative impact on ethanol production. The stress of producing PduA* is also reflected in decreased chlorophyll *a* and carotenoid levels (Figure S4). Based on our data, one can speculate that higher order structure assemblies (e.g. enzyme incorporation into the nanofilament) or direct EP‐dependent enzyme co‐localisation may aid synthetic metabolon formation. Our data point in this direction by showing that EP‐fusion of Pdc and Adh enzymes is sufficient to observe higher ethanol yields and a higher ethanol/acetaldehyde ratio (Figure S5). However, this is not sufficient evidence to determine whether the enzymes indeed co‐localise or whether PduA* still forms nanofilaments or other higher order structures (e.g., sheets) [31] in the presence of EP‐enzyme fusions. Further experimental evidence will be needed to answer these questions. Ultimately, a simple peptide fusion was sufficient to decrease the metabolic burden, enhance Pdc, improve ethanol‐to‐acetaldehyde ratios, and ultimately, increase ethanol titres. Future engineering attempts should focus on fine tuning the pathway stoichiometry to avoid acetaldehyde accumulation under enhanced production conditions.

The BMC‐derived tools presented here allowed us to achieve a peak ethanol titre of 1.31 g L^−1^ corresponding to a daily productivity of 0.187 g L^−1^ d^−1^. Therefore, these EPs show promise to be combined with established metabolic engineering strategies to push ethanol titres beyond the 1 g L^−1^ d^−1^ achieved to date in cyanobacteria (reviewed in [42]). These include knocking out competing pathways such as those for glycogen and polyhydroxybutyrate synthesis (0.193–0.987 g L^−1^ d^−1^) [43, 44, 45, 46], stimulation of CO_2_ fixation (0.060–0.100 g L^−1^ d^−1^) [47, 48], remodelling of carbon flow (0.248 g L^−1^ d^−1^) [49], and increase of native pyruvate pools (0.347 g L^−1^ d^−1^) [50].

In conclusion, we presented here a nanotechnology approach for improving protein stability and potentially also co‐localisation in Se2973 and Se7942. In conjunction with other strategies, this holds the promise to further enhance ethanol production in cyanobacteria. Beyond ethanol production, these EP tools will likely be useful for expression and tuning of other biosynthetic pathways and contribute to future efforts in cyanobacterial strain engineering.

## 4 Experimental Procedures

### 4.1 Cyanobacterial Strains and Cultivation

The strains *Synechococcus elongatus* PCC 7942 (Se7942, obtained from the Pasteur Culture Collection of Cyanobacteria) and *Synechococcus elongatus* UTEX 2973 (hereafter Se2973, obtained as a kind gift from Himadri Pakrasi) were used in this study. Strains were maintained on BG‐11 medium [51] supplemented with 10 mM 2‐tris(hydroxymethyl)‐methyl‐2‐amino 1‐ethanesulphonic acid (TES), pH 8.0 and 1.5% (w/v) Kobe I agar (Carl Roth). Plates were illuminated with approximately 50 μmol photons m^−2^ s^−1^ of white light and kept at 30 °C. Liquid cultures were grown in P4‐TES CPH medium [52, 53]. Strains with NS1 genomic integrations (pAS016, pAS017, and pAS018, Table S1) were supplemented with 10 µg mL^−1^ kanamycin except in the main experiments where antibiotics were omitted. Strains were supplemented with 25 µg mL^−1^ spectinomycin if the self‐replicative plasmids pDF‐trc or pEG001 were present (Table S1).

### 4.2 Plasmid Generation

Constructs were assembled by NEBuilder HiFi DNA assembly using the respective master mix (New England Biolabs) with *E. coli* NEB 5‐alpha as the cloning host. Linear DNA fragments were amplified using Q5 Hot Start High‐Fidelity polymerase (New England Biolabs). An overview of plasmids used in this study is given in Table S1, and primers used for assembly are specified in Table S2. Correct assembly of the plasmids was confirmed by Sanger sequencing (Eurofins Genomics). To generate the constructs, an empty vector (pAS016) was first generated using pBR322 as a backbone, whereby the tetracycline resistance cassette was replaced with 1.5 kb flanking regions for NSI, two adjacent restriction sites for SalI and BsrGI, the terminator BBa_BB0015 and a kanamycin cassette. Subsequently, pAS016 was digested with SalI and BsrGI restriction enzymes for the integration of *pdc* from *Zymomonas mobilis* and *adh* from *Synechocystis* sp. PCC 6803 under the control of the *trc* promoter (pAS017). Plasmid pAS018 was built with a similar strategy, but with the P18 EP (AA sequence: MNTSELETLIRNILSEQL) [54, 55] fused to Pdc and the D18 EP (AA sequence: MEINEKLLRQIIEDVLSE) [54, 55] fused to Adh. Both EPs were fused with two tandem Gly_4_Ser linkers to the N‐terminus of the enzymes. Additionally, all enzymes were fused with a C‐terminal HA‐tag. The operon was codon‐optimised for expression in Syn6803 and custom‐synthesised by GenScript. The gene for the scaffold protein PduA* was expressed on the plasmid pEG001 using the pDF‐trc [33] backbone as described previously [16].

### 4.3 Generation of Cyanobacterial Mutants

Constructs were introduced via triparental mating as previously described [16]. Once colonies appeared on selection plates, Se2973 and Se7942 strains transformed with pAS016, pAS017 and pAS018 were analysed via PCR to screen for genomic construct integration (Figure S1). To verify integration of pAS016, the primers UTEX2973_NSI_F (5′‐ACG GGG AAC ACA TTC GGC G‐3′) and NS1‐7942‐R (5′‐AAG GGA CCC AAC GGC TGG‐3′) were used (Figure S1A). For pAS017 and pAS018, the reverse primer was replaced with LacIq_R (5′‐CGC CAT CGC CGC TTC C‐3′) (Figure S1B). For both PCRs, Phusion High‐Fidelity DNA Polymerase (New England Biolabs) was used with the following cycling conditions: 98 °C for 10 min; 30 cycles of 98 °C for 10 s, 66 °C for 30 s, 72 °C for 1.5 min; 72 °C for 10 min. Strains were restreaked on increasing concentrations of antibiotics until full segregation was achieved. Once fully segregated, strains were conjugated via triparental mating with the pDF‐trc empty vector or the pduA*‐containing pEG001. The transformants were screened for the presence of the plasmids using primers SmR_R (5′‐CCA ACT ACC TCT GAT AGT TGA GTC‐3′) and trc‐R [53] (5′‐ATC AGG CTG AAA ATC TTC TC‐3′) with cycling conditions as described above, except with an annealing temperature of 60 °C (Figure S1C).

### 4.4 Ethanol Production Time Course

For analysis of ethanol production over time, the strains were cultivated in a Multi‐Cultivator MC 1000‐OD photobioreactor (Photon Systems Instruments) in P4‐TES CPH medium. Strains transformed with the pDF‐trc or pEG001 plasmids were supplemented with 25 μg mL^−1^ spectinomycin. Pre‐cultures were cultivated at 30 °C with a light ramp in the first 48 h from 80 to 300 μmol photons m^−2^ s^−1^ for Se7942 strains and from 80 to 500 μmol photons m^−2^ s^−1^ for Se2973 strains, followed by constant illumination and were then used to inoculate main cultures to a cell count of approximately 8 × 10^7^ cells per mL. The main cultures were cultivated at 30 °C with the same light ramps. Main cultures were induced with 0.5 mM isopropyl‐D‐1‐thiogalactopyranoside (IPTG) at Time 0 and cultivated for 6 or 7 days for Se2973 and Se7942, respectively. Due to the altered morphologies (Figure S3) and pigment content (Figure S4) observed in the engineered strains, cell counts were chosen to follow growth progression of the cultures. Cell counts were performed with a CASY Cell Counter (OLS OMNI Life Sciences) with the aggregation factor correction turned off. Due to the filamentous phenotype of the PduA* strains, the accuracy of the cell counts was verified by manual counting with a Thoma chamber (Figure S6). To estimate growth rates and carrying capacities of the growth data, logistic curves were fit with GraphPad Prism 11.0.0 using the nonlinear fit analysis with the robust regression fitting method and each replicate Y value considered as an individual point. The remaining settings were left with their default values. To determine pigment content over time, the chlorophyll *a* and carotenoid content of samples were estimated as previously described [56] with the following modifications: Samples were centrifuged at 10 000 × *g* for 5 min at 4 °C before measurement.

### 4.5 Ethanol Quantification

Ethanol was detected in culture supernatants via an enzymatic assay based on [57] with small modifications. The assay was conducted in microtiter plates with 180 µL reactions in 100 mM potassium phosphate buffer (pH = 6) containing the following components: 60 µL cell‐free supernatant, 100 µg of 2,2′‐azino‐bis(3‐ethylbenzothiazoline‐6‐sulphonic acid) (ABTS; Sigma‐Aldrich) from a 10 mg mL^−1^ stock, 10 µL of horseradish peroxidase (Sigma‐Aldrich) containing 0.25 units, and 100 µL of alcohol oxidase (Sigma‐Aldrich) containing 0.5 units. When necessary, samples were diluted with growth medium to keep within the range of 0.25–4 mM ethanol. The absorbance of oxidised ABTS was measured after 4 min incubation at 420 nm using an Agilent BioTek Synergy H4 Hybrid Reader and values were compared with an ethanol standard curve.

### 4.6 SDS‐PAGE and Immunoblot Analysis

Cell pellets equivalent to an optical density at 750 nm = 5 were resuspended in 250 µL lysis buffer (5% glycerol and 1% Triton X‐100 in 20 mM Tris‐HCl pH 7.5) [53]. Zirconium oxide beads (0.15 mm) were added, and cells were disrupted using a Bullet Blender Storm tissue homogeniser (Next Advance) for three cycles of 5 min at level 12. The samples were subsequently centrifuged at 10 000 × *g* for 10 min at 4 °C to sediment cell debris and the supernatant was then used for further analysis. Proteins were quantified using ROTI Nanoquant (Carl Roth) following manufacturer's instructions. SDS‐PAGE was performed as previously described [58] and proteins blotted onto 0.2 µm nitrocellulose membranes using the Trans‐Blot Turbo system (Bio‐Rad Laboratories) using the setting for mixed molecular weights (2.5 A, 25 V, 7 min). Treatment, development and imaging of the blots was done as previously described [58]. Densitometric analysis was performed using Image Lab 6.0.1 software (Bio‐Rad Laboratories).

### 4.7 Pyruvate Feeding Experiment

Strains were grown in 20 mL P4‐TES CPH medium supplemented with their respective antibiotics, where necessary, and induced with 0.5 mM IPTG at time zero. Cultures were bubbled with 3% (v/v) CO_2_‐supplemented air and cultivated with stepwise increasing light intensities from 90 to 300 μmol photons m^−2^ s^−1^ or 500 μmol photons m^−2^ s^−1^ for Se7942 and Se2973, respectively. To test the effect of pyruvate feeding, cultures were supplemented with 2 mM pyruvate every 24 h. Acetaldehyde levels were determined using a 3‐methyl‐2‐benzothiazolinone hydrazone (MBTH) assay as previously described [59, 60] with minor modifications. Assays were performed in 358 µL containing 93 µL of water, 143 µL of 100 mM potassium citrate (pH 3.6), 50 µL of appropriately diluted sample and 72 µL of 0.1% MBTH. Assays mixtures were incubated at 37 °C for 30 min prior to measurement of absorbance at 305 and 350 nm. In the pyruvate feeding experiments, the reaction product of MBTH with pyruvate (*λ*_max_ = 350 nm) partially overlapped with the spectrum of the acetaldehyde product (*λ*_max_ = 305 nm). Therefore, the following equation based on the additivity principal of the Beer–Lambert law was used to to determine the concentration of aldehyde in the treatments with added pyruvate:

$$\text{Acetaldehyde}\;\left(\mathrm{mM}\right)\;=\;\frac{A_{305}*m_{P350}\;-\;A_{350}*m_{P305}}{m_{A305}*m_{P350}\;-\;m_{A350}*m_{P305}}$$

*A*_305_ and *A*_350_ correspond to the absorbances of the assay mixtures at 305 and 350 nm, respectively, and *m*_A305_, *m*_A350_, *m*_P305_, and *m*_P350_ are the slopes derived from the calibration curves of acetaldehyde and pyruvate measured at 305 and 350 nm. The calibration curves for acetaldehyde and pyruvate were performed in P4‐TES CPH medium to account for any potential background signals derived from the growth medium.

### 4.8 Microscopy

Liquid cultures for microscopy were grown in 20 mL P4‐TES CPH medium at 30 °C in glass tubes illuminated with approximately 50 μmol of photons m^−2^ s^−1^ and bubbled with 3% (v/v) CO_2_‐supplemented air. Cultures were induced upon inoculation from agar plates with 0.1 mM IPTG and supplemented with 25 µg mL^−1^ spectinomycin. After 48 h, microscopy was performed using a LSM 880 microscope (Carl Zeiss) with the 488 nm laser line produced by an argon multiline laser (11.5 mW). Images were taken by a 40x objective (Plan‐Apochromat 40_/0.8). Micrographs were processed with the ZEN (black edition) software (Carl Zeiss).

## Funding

This work was supported by the Deutsche Forschungsgemeinschaft (DFG, German Research Foundation), SFB 1127 ChemBioSys, project number 239748522.

## Conflicts of Interest

The authors declare no conflicts of interest.

## Supporting information

Supplementary Material

## Data Availability

All data supporting the findings of this study are available within the paper and its Supplementary Information or will be made available by the corresponding author upon reasonable request.
