# Supplementary material for: Employing Nanotechnology to Enhance Ethanol Production in Two Synechococcus elongatus Strains
Source: Chembiochem. 2026 Jul 24;27(14):e70480. doi: 10.1002/cbic.70480 (PMC13398025; doi:10.1002/cbic.70480)
Supplement: Supplementary file 1 — Supplementary Material [file CBIC-27-e70480-s001.pdf]

Supplementary Information

Employing nanotechnology to enhance ethanol production in two *Synechococcus elongatus* strains

Alexandra M. Schirmacher[1], David A. Russo[2], Alexandra C. U. Furch[1] and Julie A. Z. Zedler[1*]

[1] Synthetic Biology of Photosynthetic Organisms, Matthias Schleiden Institute for Genetics, Bioinformatics and Molecular Botany, Friedrich Schiller University Jena, 07743 Jena, Germany
[2] Bioorganic Analytics, Institute for Inorganic and Analytical Chemistry, Friedrich Schiller University Jena, 07743 Jena, Germany

*Correspondence:
Email: julie.zedler@uni-jena.de

Overview of content:

Figure S1 Colony PCR of engineered strains 2
Figure S2 Ethanol titres at different cultivation temperatures in Se2973. 2
Figure S3 Light microscopy of cells showing PduA* phenotype. 3
Figure S4 Pigment content of engineered strains over time 4
Figure S5 Pyruvate supplementation experiment in Se2973 and Se7942. 5
Figure S6 Comparison of cell counting methods. 6
Table S1 Plasmids used in this study. 7
Table S2 Primers used for construct assembly. 7
References 8

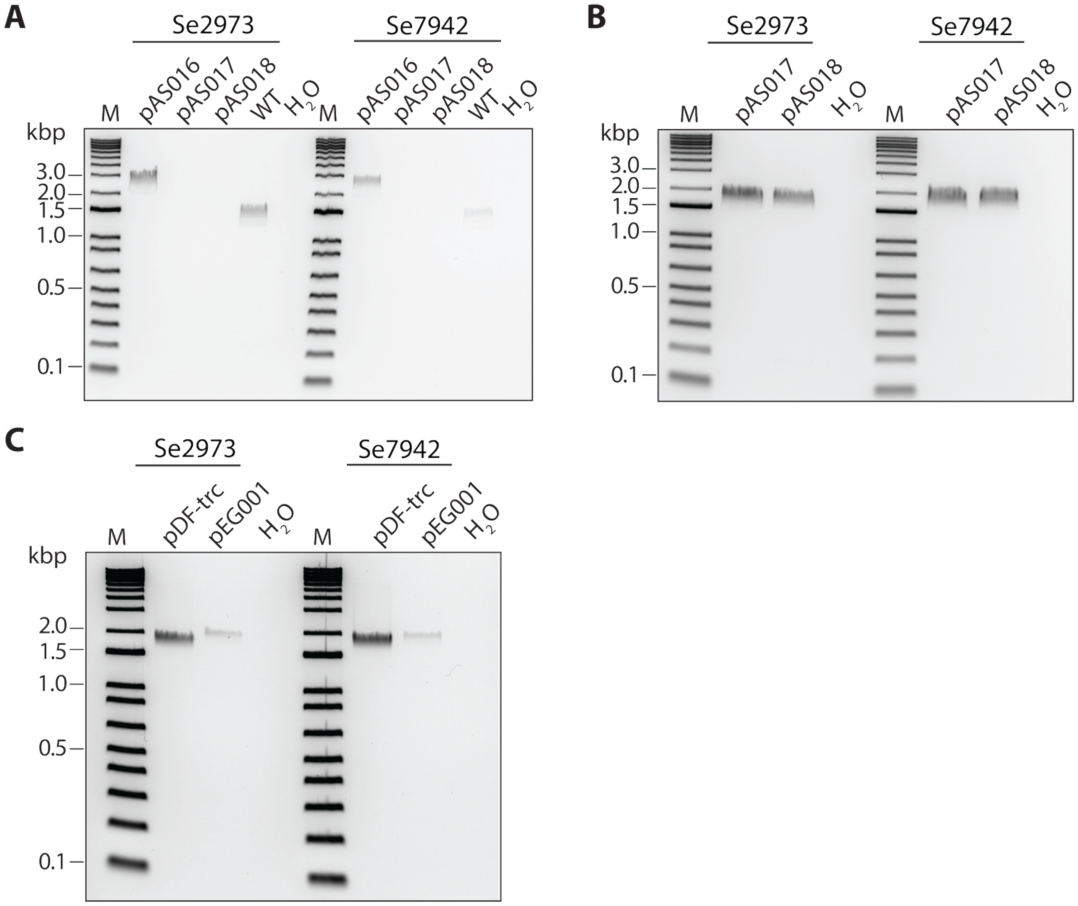

Figure S1. Segregation of engineered Se2973 and Se7942 strains confirmed by colony PCR. **A** Amplification of NS1 genomic region with primers UTEX2973_NSI_F and NS1-7942_R. **B** Amplification of integrated ethanol operon in NS1 site with primers UTEX2973_NSI_F and LacIq_R. **C** Confirmation of pDF-trc (background strain with pAS016 integrated) and pEG001 (background strain with pAS018 integrated) self-replicative plasmid presence using primers SmR_R and trc-R.

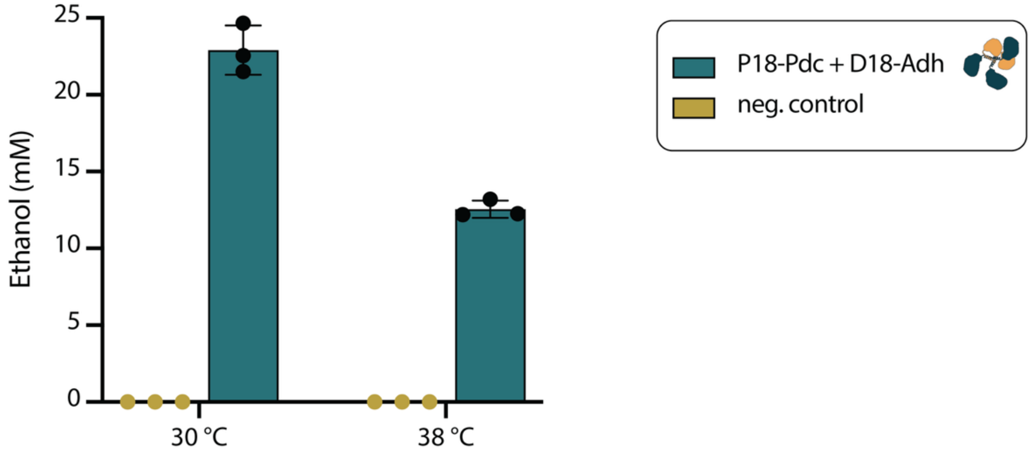

Figure S2. Comparison of ethanol titres detected in the medium of Se2973 engineered strains cultivated at 30 °C and 38 °C. Replicates: n = 3, error bars ± SD.

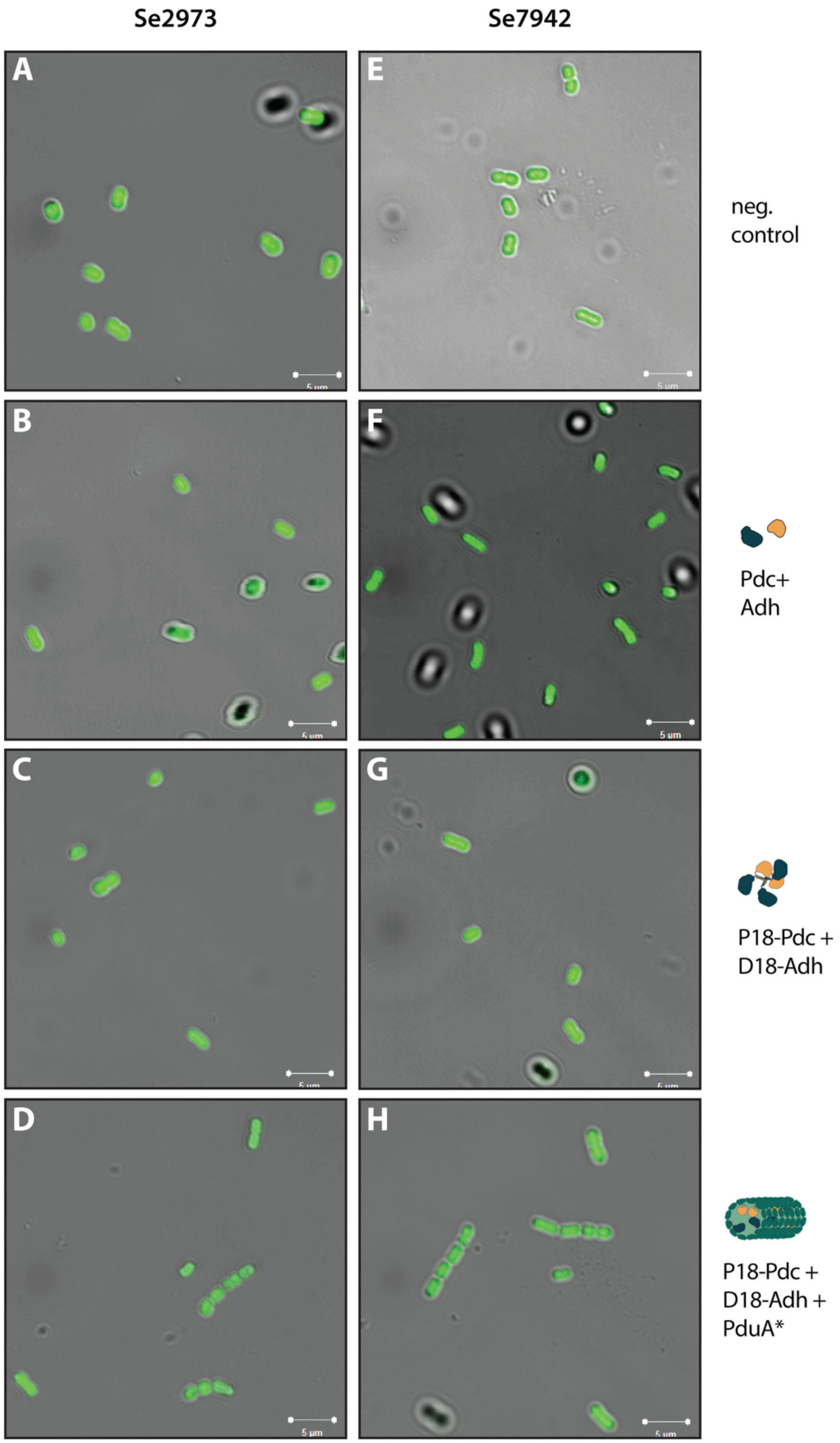

Figure S3. Phenotypic differences observed in different engineered strains of Se2973 (**A–D**) and Se7942 (**E–H**). All strains are similar in cell size and show active division. A filamentous phenotype of *pduA** expressing Se2973 (**D**) and Se7942 (**H**) strains indicating interference of PduA* with cell septation. Cultures imaged after 2 days of cultivation. Overlay of chlorophyll *a* autofluorescence and bright field images. Scale bar: 5 µm.

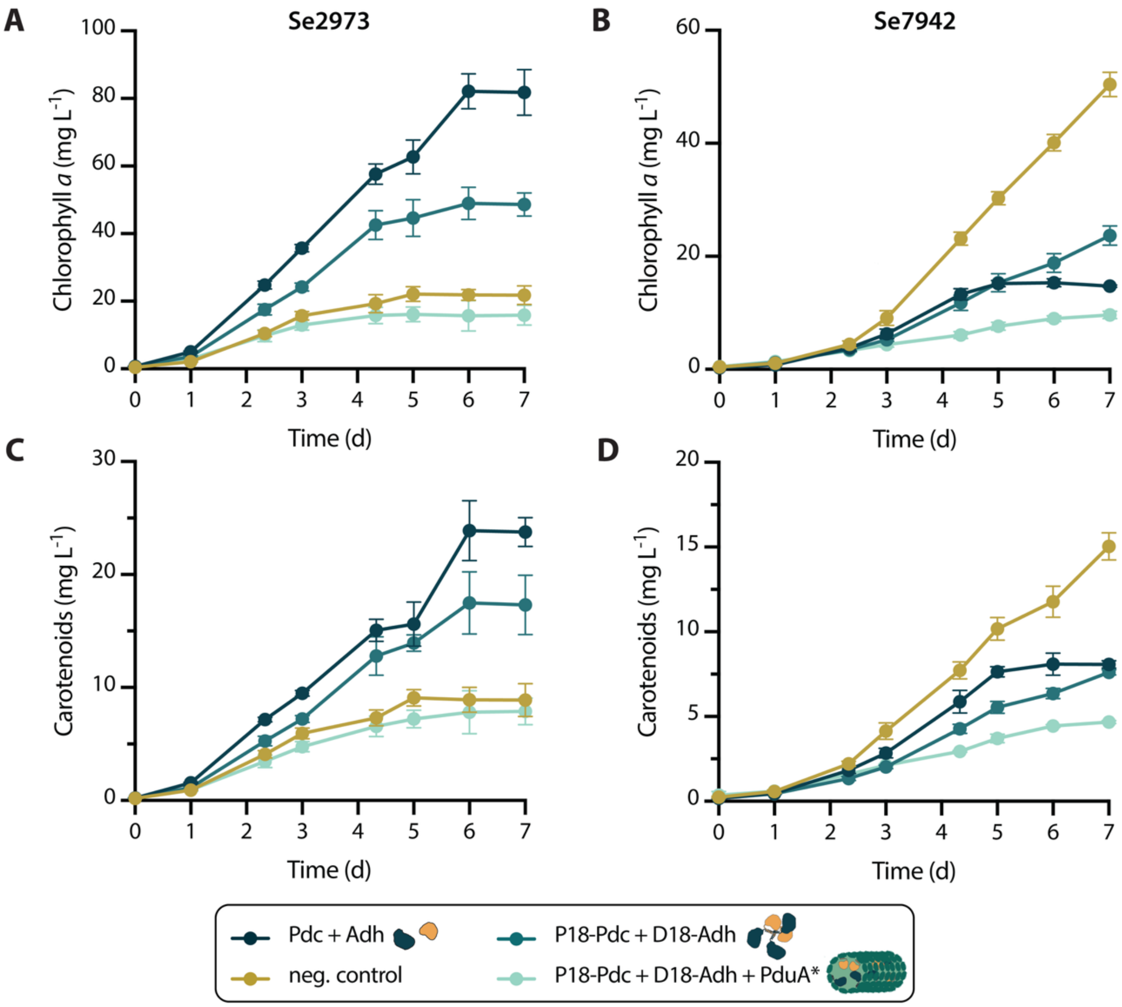

Figure S4. Estimation of pigment content in Se2973 (A, C) and Se7942 (B, D) strains over time. **A, B** Estimation of chlorophyll *a* content in mg L^{-1}. **C, D** Estimation of total carotenoid content in mg L^{-1}. Replicates: n = 4, error bars: ±SD.

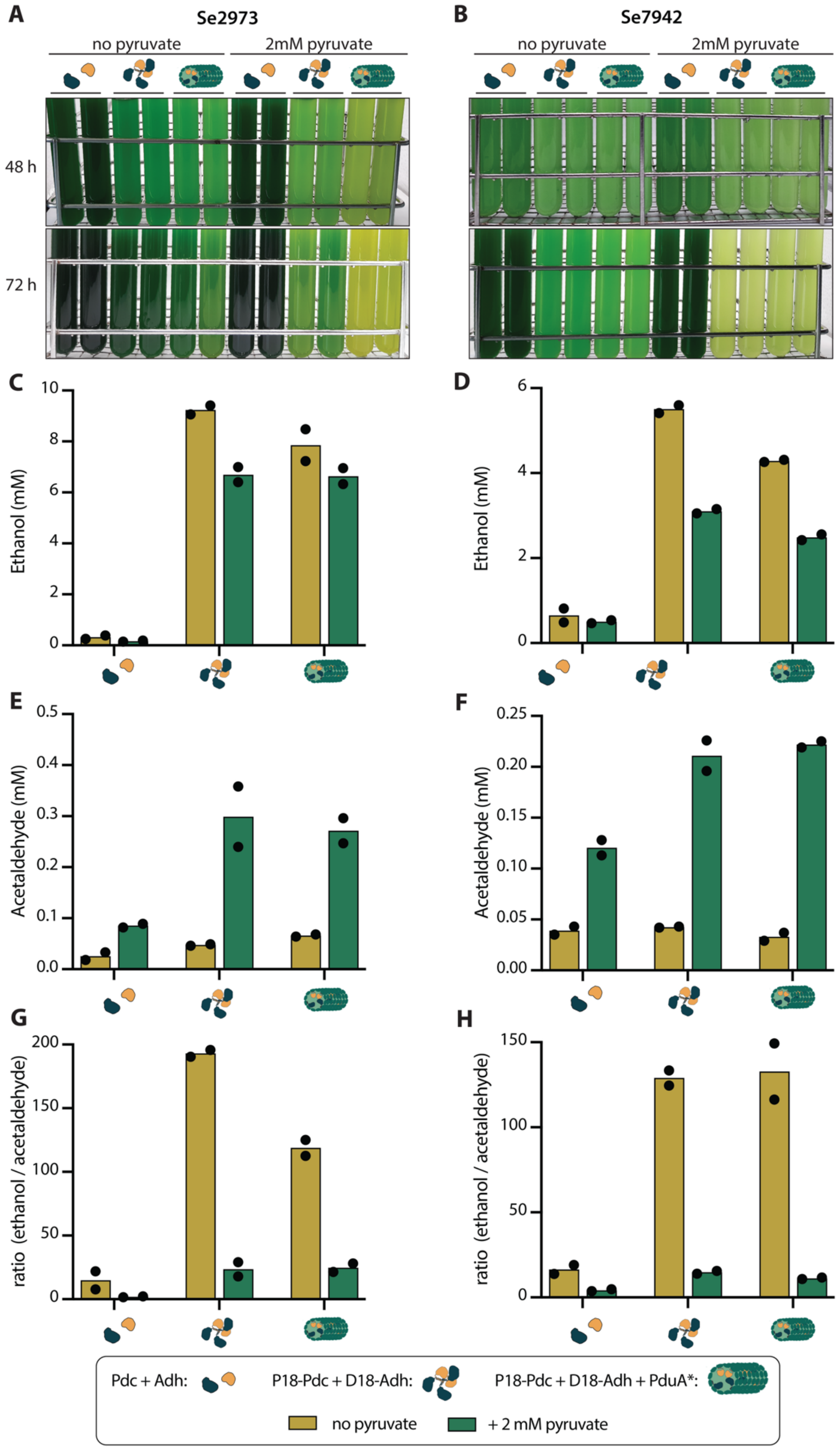

Figure S5. Supplementation of the precursor pyruvate in Se2973 (A, C, E, G) and Se7942 (B, D, F, H) for ethanol production. **A, B** Visual documentation of growth of Se2973 (A) and Se7942 (B) strains after 48 and 72 h of cultivation with and without daily 2 mM pyruvate supplementation. **C, D** Total ethanol production after 48 h of incubation. **E, F** Acetaldehyde concentration after 48 h of incubation. **G, H** Ratio of ethanol per acetaldehyde after 48 h. All data shown with (green bars) and without (golden bars) daily 2 mM pyruvate supplementation measured in cell-free supernatant of cultures of Se2973 and Se7942 strains. Replicates: n = 2.

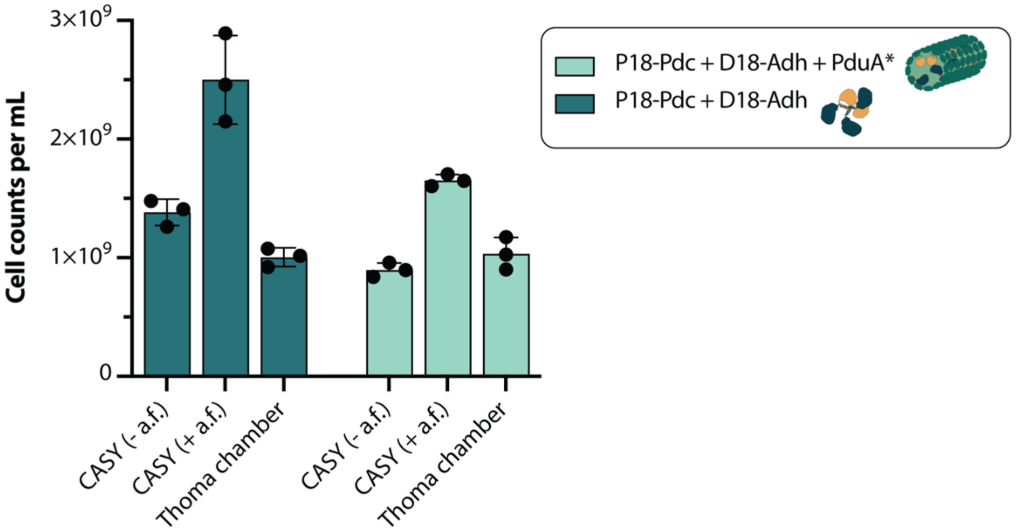

Figure S6. Comparison of cell counts using a CASY cell counter with (+) and without (–) the aggregation factor (a.f.) correction and manual counting using a Thoma counting chamber. Replicates: n = 3, error bars ± SD.

Table S1. Plasmids used in this study for engineering Se2973 and Se7942.

Plasmid	Details	Strains generated using the respective plasmid	Origin of this plasmid
pDF-trc	Replicative plasmid; selection marker: streptomycin resistance cassette.	Negative control	[1]
pEG001	Promoter Ptrc, *pduA**, Terminator T_{rrnB}; backbone: pDF-trc.	P18-Pdc + D18-Adh + PduA*	[2]
pAS016	Integrative plasmid for neutral site 1 (NSI) with Terminator T_{BB0015}, selection marker: KanR; backbone: pBR322.	Negative control	this study
pAS017	Integrative plasmid for neutral site 1 (NSI) Ptrc, Pdc-HA, Adh-HA, TBB0015, KanR, NSI DOWN flanking region; backbone: pBR322.	Pdc + Adh	this study
pAS018	Integrative plasmid for neutral site 1 (NSI) with Ptrc, P18-Pdc-HA, D18-Adh-HA, TBB0015, Kan^R, NSI DOWN flanking region; backbone: pBR322.	P18-Pdc + D18-Adh<br><br>P18-Pdc + D18-Adh + PduA*	this study

Table S2. Primers used for construct assembly.

Primer	Sequence (5'-3')	target plasmid
pBR322_F	ATG GAA GCC GGC GGC	pAS016
pBR322_R	GAA TTC TTG AAG ACG AAA GGG CCT CG	pAS016
UTEX2973_NSI UP_ovF	CGT CTT CAA GAA TTC GCT GCT AGC GAC GAG AGC AC	pAS016
UTEX2973_NSI UP_ovBsrTerR	GTG TAC ATA ATA AGT CGA CTC GAG TCC CTG CTC GTC ACG	pAS016
TerBBa BB0015_ovBsrF	TCG AGT CCC TGC GTC GAC TTA TTA TGT ACA CCA GGC ATC AAA TAA AAC GAA AGG	pAS016
KanR_ovNSIR	GCC CGC GAC ATC TTC CTG CTC CAG AAG CGG ATC CCT CGA GTC CCG	pAS016
UTEX2973_NSI DOWN_ovF	GAC GGG ACT CGA GGG ATC CGC TTC TGG AGC AGG AAG	pAS016
UTEX2973_NSI DOWN_ovR	GCC GCC GGC TTC CAT AGG CCT CAA GTC CGC TCC GAG	pAS016
Ptrc_ovNSIF	CGA GCA GGG ACT CGA GTC GAT TAC GTT GAC ACC ATC GAA TGG	pAS017+ pAS018
Adh_ovTerR	TTT CGA CTG AGC CTT TCG TTT TAT TTG ATG CCT GGT GTA CAG CCA AAA CAG CCA AGC TTC	pAS017+ pAS018

References

[1] F. Guerrero, V. Carbonell, M. Cossu, D. Correddu, P. R. Jones, “Ethylene Synthesis and Regulated Expression of Recombinant Protein in *Synechocystis* sp. PCC 6803” *PLoS One* **2012**, *7*, e50470.

[2] J. A. Z. Zedler, A. M. Schirmacher, D. A. Russo, L. Hodgson, E. Gundersen, A. Matthes, S. Frank, P. Verkade, P. E. Jensen, “Self-Assembly of Nanofilaments in Cyanobacteria for Protein Co-localization” *ACS Nano* **2023**, *17*, 25279–25290.
